# Validation of the Dutch-language version of Nurses’ Moral Courage Scale

**DOI:** 10.1177/0969733020981754

**Published:** 2021-01-11

**Authors:** Olivia Numminen, Kasper Konings, Roelant Claerhout, Chris Gastmans, Jouko Katajisto, Helena Leino-Kilpi, Bernadette Dierckx de Casterlé

**Affiliations:** 60654 University of Turku, Finland; 26657 KU Leuven, Belgium; 60654 University of Turku, Finland; 26657 KU Leuven, Belgium

**Keywords:** Belgium, Dutch language, instrument validation, moral courage, nurses, psychometric evaluation

## Abstract

**Background:**

Moral courage as a part of nurses’ moral competence has gained increasing interest as a means to strengthen nurses acting on their moral decisions and offering alleviation to their moral distress. To measure and assess nurses’ moral courage, the development of culturally and internationally validated instruments is needed.

**Objective:**

The objective of this study was to validate the Dutch-language version of the four-component Nurses’ Moral Courage Scale originally developed and validated in Finnish data.

**Research design:**

This methodological study used non-experimental, cross-sectional exploratory design.

**Participants and research context:**

A total of 559 nurses from two hospitals in Flanders, Belgium, completed the Dutch-language version of the Nurses’ Moral Courage Scale.

**Ethical considerations:**

Good scientific inquiry guidelines were followed throughout the study. Permission to translate the Nurses’ Moral Courage Scale was obtained from the copyright holder, and the ethical approval and permissions to conduct the study were obtained from the participating university and hospitals, respectively.

**Findings:**

The four-component 21-item, Dutch-language version of the Nurses’ Moral Courage Scale proved to be valid and reliable as the original Finnish Nurses’ Moral Courage Scale. The scale’s internal consistency reliability was high (0.91) corresponding with the original Nurses’ Moral Courage Scale validation study (0.93). The principal component analysis confirmed the four-component structure of the original Nurses’ Moral Courage Scale to be valid also in the Belgian data explaining 58.1% of the variance. Confirmatory factor analysis based on goodness-of-fit indices provided evidence of the scale’s construct validity. The use of a comparable sample of Belgian nurses working in speciality care settings as in the Finnish study supported the stability of the structure.

**Discussion and conclusion:**

The Dutch-language version of the Nurses’ Moral Courage Scale is a reliable and valid instrument to measure nurses’ self-assessed moral courage in speciality care nursing environments. Further validation studies in other countries, languages and nurse samples representing different healthcare environments would provide additional evidence of the scale’s validity and initiatives for its further development.

## Introduction

In the discussion concerning nurses’ ethical competence, the concept of moral courage has gained increasing interest.^1–5^ It is seen as a means to strengthen and empower nurses in their ethical decision-making and its implementation within a multi-professional healthcare team, as well as to offer alleviation to moral distress commonly experienced by nurses.^6–10^ Furthermore, nurses’ morally courageous action has also been seen as a contribution to patient safety and quality nursing care^11–13^. However, understanding and assessing nurses’ moral courage has been largely based on anecdotal evidence rather than on scientific evidence. Developing instruments to measure moral courage, particularly to nursing contexts and valid also internationally, is one way of increasing scientific evidence of nurses’ moral courage and thus to find reliable justifications for means to advance nurses’ moral courage. The Nurses’ Moral Courage Scale (NMCS^©^)^14^ has been developed in Finland to measure nurses’ self-assessed level of moral courage. The aim of this study was to assess the psychometric validity of the Dutch-language version of the NMCS in nursing context in Flanders (Belgium).

## Background

Moral courage has been defined as courage in the context of a moral issue.^15^ It stems from an individual’s moral stand involving deliberation and careful thought, referring to moral reasoning. It is an individual’s ability to use inner principles to do what is right and good for others, regardless of the threat of negative consequences to self. Based on the contemporary view, a motivating factor in moral courage is acting for and defending a collective good.^16–19^ Thus, moral courage is a form of prosocial behaviour with high social costs and no direct reward for the actor.^20^ Moral courage does not mean absolute fearlessness but an ability to overcome fear.^21^ Moral courage is ‘the golden mean’ between rashness and cowardice, rashness indicating thoughtlessness and cowardice, irrational fear.^22^ In nursing, moral courage refers to the nurse’s ability to rationally defend professional and personal ethical principles and values, and to act accordingly despite the anticipated or real adverse consequences of such actions to self.^14,23–25^ Lack of moral courage undermines nurses’ integrity as autonomous moral agents.^6,26–28^

Nurses encounter ethical problem situations daily. These arise when different values related to care are in conflict or the conceptions of the primacy of values are in conflict with each other.^29^ To recognize these situations, nurses need ethical sensitivity and ability to choose the solution that best enhances their patients’ comprehensive well-being.^30,31^ This in turn requires ethical deliberation and ability to make ethical decisions.^32,33^ But mere deliberation and ability to make decisions do not suffice, the nurse has to be able also to act according to her or his decisions to fulfil the goal of ethical nursing care.^1,30^ However, in the traditionally hierarchal healthcare organizations, nurses have often felt themselves uncertain, silenced and banished from ethical decision-making.^33–37^ This has manifested as moral distress and a lack of moral courage ^36,38–40^ as well as nurses’ conventional and conformist decision-making by adhering to socially accepted rules, norms and expectations, rather than courageously relying on their own principles and professional ethical values.^41,42^

Thus, to strengthen their role in ethical decision-making and their ability to act according to their professional and personal ethical principles and values, nurses need moral courage.^5,43,40^

Nurses’ ethical competence, including moral courage as one of its elements, has been defined as a key characteristic in contributing to high-quality and safe patient care.^11– 13^ The ICN Code of Ethics for Nurses (2012) goes as far as stating the following: ‘The nurse contributes to an ethical organizational environment and challenges unethical practices and settings’, which requires moral courage of the nurse, thus entailing intervention in the norm violations of the more powerful.^11,44^

As an individual human characteristic, moral courage has been studied particularly in the field of social psychology.^16,17,45,46^ In nursing, research on moral courage has been fairly scarce, but it has increased in the last 10 years. The majority of studies have used qualitative research designs,^32,47–58^ thus offering generalizable theoretical or inferential knowledge of moral courage, but not lending itself to measure the level and determinants of moral courage.

The other studies have been literature reviews ^24,59,60^ and concept analyses.^9,61^ Only a few studies have used quantitative research designs to explore nurses’ moral courage and associated variables, of which just one focused on the validation of a moral courage scale.^14,62,63^

Discussion of nurses’ ethical competence has instigated a discussion about the need to measure and assess nurses’ ethical competence including moral courage and to provide research-based knowledge of it in wider contexts with more generalizable findings (ProComp, https://sites.utu.fi/nursingscienceresearchprogrammes/pedagogic/procompnurse/). Development and validation of relevant instruments adaptable to various countries and languages, healthcare cultures and contexts would contribute to providing evidence-based knowledge of issues which all call for ethical competence and moral courage, such as globalization and migration in nursing, nurses’ contribution to solving global health issues, COVID-19 pandemic as a recent example, promotion of ethical nurse leadership and many others.^64^ Such instruments would also serve many goals set by the Council of Europe concerning higher education in terms of international harmonization and standardization of nursing practices and education.^13,65^ Internationally valid instruments would enable evaluation and comparability of nurse education programmes and practices leading to a greater common understanding of the concept of moral courage in provision of ethical nursing care. To achieve these goals, international collaborative research has been regarded important and that nursing practice, leadership and education rest on evidence-based knowledge.^66–68^ However, particularly in the field of nursing ethics research, there is a scarcity of standardized and validated instruments to pursue the set goals.^10^

Only a few validated instruments have been developed to measure moral courage. Sekerka et al.’s^45^ instrument was originally designed to measure moral courage in business organizations, and Martinez et al.’s^69^ instrument measures moral courage of medical residents and interns. In nursing, Dinndorf-Hogenson^70^ developed a questionnaire to measure perioperative nurses’ moral courage from the perspective of patient safety.^70,71^ However, there is no indication of this instrument’s psychometric validation. To contribute to the demand of validated instruments in nursing ethics research, the NMCS was developed to measure nurses’ self-assessed level of moral courage. The instrument has been validated in Finland in the Finnish healthcare context.^14^ The availability of internationally validated instruments will enable researchers to measure and assess nurses’ moral courage in a variety of caring contexts and cultures. This evidence-based knowledge may contribute to a greater common understanding of moral courage in nursing and how to support nurses in showing courage in challenging ethical situations.

### The aim of the study

The aim of this study was to validate the Dutch-language version of the four-component NMCS in Flanders, Belgium.

## Methods

### Design, sample and setting

A methodological non-experimental, cross-sectional exploratory research design was used to study a convenience sample of registered nurses working in various care departments in two hospitals, one in an academic setting and one in a regional setting in Flanders, Belgium. The departments were selected in agreement with the researchers and the hospital managements. The departments comprised a representative sample of nurses working in a variety of care contexts in specialized care environments. Eligibility to participate was as follows: The nurse (a) had to be a professional nurse and to have a diploma of a graduated nurse as the educational minimum, (b) had to have a sufficient command of the Dutch language to be able to complete the NMCS and (c) had to work presently in the participating hospital. Based on statistical power analysis,^72^ at a significant level of p ≤ 0.05 and 95% confidence level, the sample size estimation was 559 nurses.

### Measure

The NMCS measures nurses’ self-assessed level of moral courage. The item content of the NMCS is based on a concept analysis^9^ and related literature. The original scale has 21 items distributed across four dimensions (sub-scales) of moral courage named as (a) *compassion and true presence* (five items), (b) *moral responsibility* (four items), (c) *moral integrity* (seven items) and (d) *commitment to good care* (four items).^14^ Self-assessed moral courage is measured using a 5-point Likert-type scale, the total NMCS score rendering the mean of the scores of the 21 items. In addition, the respondents are asked to assess their overall moral courage on a visual analogue scale (VAS) 0–10, in which 0 indicates never and 10 indicates always acting morally courageously. Response to 10 socio-demographic variables which are age, gender, highest educational degree, work experience, work role, hospital department where working, knowledge base in healthcare ethics, way of acquiring this knowledge base, participation in development of healthcare ethics, and frequency of care situations needing to show moral courage is also asked.

The original version of the NMCS was developed and validated in a nurse sample working in speciality care units in a large university hospital in Finland. The scale’s internal consistency reliability value using Cronbach’s alpha for the total scale was 0.93 and for the sub-scales was as follows: *compassion and true presence*, 0.81; *moral responsibility*, 0.81; *moral integrity*, 0.82; and *commitment to good care* 0.73. Initial construct validity of the Finnish-language NMCS was assessed using confirmatory factor analysis (CFA). Several criteria were used to examine the goodness-of-fit of the construct, and the results of which are shown in Table 1. All criteria (process capability index (CP), comparative fit index (CFI), standardized root mean square residual (SRMR) and root mean square error of approximation (RMSEA)) confirmed the goodness-of-fit of the construct (n = 482).^14^

**Table 1.** Goodness-of-fit indices for the hypothesized model/Model 1 of the Finnish NMCS (N = 482).

Model	χ^2^	p value	df	CFI	TLI	AIC	BIC	SRMR	RMSEA	p < 0.05
	1.107	0.5748	2	1.000	1.002	2241.05	2291.82	0.004	0.000	0.830

χ^2^, p value: non-significant chi-square statistics; df: degree of freedom; CFI: comparative fit index; TLI: Tucker–Lewis index; AIC: Akaike information criterion; BIC: Bayesian information criterion; SRMR: standardized root mean square residual; RMSEA: root mean square error of approximation.

The Dutch-language version used in this study was based on the English-language version of the scale, double-translated from the original Finnish-language scale which had been validated in Finnish data.^14^

### Translation of NMCS

Internationally recommended steps to carry out a cross-cultural adaptation of an instrument (NMCS) were followed.^73–75^ In the double-translation of the NMCS, a change was made to the socio-demographic variables adding the employment rate. Thus, the final Dutch-language version of the NMCS comprised 21 items measuring nurses’ self-assessed moral courage across four dimensions. In addition, the respondents answered 11 socio-demographic variables.

### Ethical considerations

The study followed good scientific inquiry guidelines, and all measures were carefully deliberated based on the Horizon 2020 programme established by the European Commission (https://ec.europa.eu/programmes/horizon2020/what-horizon-2020). Permission to translate and use the NMCS was obtained from the copyright holder.^14^

Ethical approval for the study was obtained from the Ethics Committee of the University of KU Leuven (MP007372), and the approval to conduct the study was obtained from the participating hospitals. Participants were informed of voluntary participation in a letter attached to the NMCS paper-and-pencil and digital form. Participant anonymity was guaranteed by using coded questionnaires, and personal responses were not shared with the participating hospital. Results of the study will be stored in an international database hosted by the University of Turku, Finland. Data storage and sharing is carried out in accordance with the General Data Protection Regulation (GDPR), EU Directive 95/46/EC and other legislation concerning data storage and privacy. Completing the questionnaire was regarded as an informed consent to participate.

### Data collection

Data were collected in February and April 2019 in an academic and a regional setting in Flanders, Belgium. The researchers contacted the managers of the hospital departments to whom the purpose of the study was explained. The researchers personally distributed the NMCS questionnaires to the participants. Both paper-and-pencil and digital questionnaires were used. The opted response rate was 50%. A total of 1352 questionnaires were distributed: in Hospital 1, 778 paper-and-pencil questionnaires, of which 350 were completed (response rate, 45.4%) and in Hospital 2, 574 digital questionnaires, of which 209 were completed (response rate, 36.3%).

### Data analysis

The data were analysed using SPSS 25.0 (IBM Corporation). The psychometric evaluation of the NMCS included Kaiser–Meyer–Olkin (KMO) measure of sampling adequacy and Bartlett’s test of sphericity to show whether the data were suitable for structure detection. To evaluate the construct validity of the scale, the forced four-component principal component analysis (PCA) with Promax and Kaiser Normalization Rotation were computed to define the structure for the scale. CFA was computed to examine the construct validity of the theoretical model based on the concept analysis and to confirm the structure of the NMCS.^76^

Internal consistency reliability was estimated using Cronbach’s alpha coefficients, the minimum value of 0.7 regarded as acceptable.^77^ Corrected item-total correlation with the minimum criteria of r = 0.30 and inter-item correlation with an acceptable value of 0.3 ≤ r ≤ 0.7 were used in item analyses. Descriptive statistics, that is, frequencies, percentages, means and standard deviations, were used in item, sub-scale and total-scale analyses.

## Results

### Respondents and descriptive statistics

A total of 559 nurses from 38 wards completed the Dutch-language NMCS yielding a response rate of 41.3%. Most participants were fairly young (44% <35 years). The majority of the respondents (84.8%) were women. About two thirds (73.5%) had a bachelor’s degree in nursing, the majority (86.9%) were working as ward nurses and about half (52.8%) were working full-time. A total of 73.2% had work experience more than 5 years.

The mean score for the NMCS total was 3.77 (SD = 0.537; range, 1.62–5.00) (Table 2). The highest mean score for a single dimension was 4.00 (SD = 0.584; range, 1.40–5.00) for *compassion and true presence*, and the lowest was 3.62 (SD = 0.638; range, 1.20–5.00) for *commitment to good care*. The detailed results of respondents’ self-assessed level of moral courage and its associations with their socio-demographics will be reported elsewhere.

**Table 2.** Descriptive statistics of sub-scales of the NMCS (N = 559).

Sub-scales	N	No. of items	Mean	SD	Min	Max	Cronbach’s alpha	Item-total r ≥ 0.3 Min–Max	Inter-item 0.3 ≤ r ≤ 0.7		
									Mean	Min–Max	%
Compassion and true presence	557	5	4.00	0.584	1.40	5.00	0.76	0.46–0.64	0.39	0.32–0.55	100
Moral responsibility	555	4	3.66	0.725	1.25	5.00	0.80	0.47–0.74	0.50	0.36–0.80	83
Moral integrity	554	7	3.78	0.594	1.86	5.00	0.81	0.38–0.64	0.37	0.18–0.55	57
Commitment to good care	552	5	3.62	0.638	1.20	5.00	0.71	0.37–0.61	0.34	0.24–0.54	50
NMCS^©^ total	545	21	3.77	0.537	1.62	5.00	0.91	0.40–0.72	0.34	0.05–0.80	54

NMCS: Nurses’ Moral Courage Scale.

### Reliability of the NMCS

Internal consistency reliability using Cronbach’s alpha coefficient was 0.91 for the NMCS total, and ranged from 0.71 to 0.81 for the four sub-scales (Table 2). Item analysis showed that in all four sub-scales, the item-total correlation exceeded the minimum value of r ≥ 0.30. Inter-item correlations showed 100% acceptability for the sub-scale *compassion and true presence*, 83% for *moral responsibility*, 57% for *moral integrity* and 50% for *commitment to good care,* and 54% for the total NMCS, based on the criteria 0.30 ≤ r ≤ 0.70.

### Construct validity of the NMCS

The forced four-factor PCA was computed for the 21-item NMCS in a sample of 559 nurses from different care settings in two hospitals. KMO measure of sampling adequacy supported the adequacy of sample size for the analysis (KMO = 0.922), and Bartlett’s test (χ^2^ = 5086, df = 210, p < 0.001) indicated that the strength of the relationship among variables is strong, thus conforming the suitability of the data (n = 559) for PCA. PCA showed that the four components accounted for 58.1% of the variance of the NMCS. The item correlations (communalities) in PCA for the NMCS ranged from 0.432 to 0.813 (Table 3).

**Table 3.** Pattern matrix of factor loadings of the NMCS items from PCA (n = 559).^a^

NMCS sub-scales and items	Components				Communalities
	1.	2.	3.	4.	
1. Compassion and true presence					
I support a suffering patient by being truly present for him or her even if…	**0.895**				0.650
I discuss the fears caused by the illness with my patient even if…	**0.770**				0.553
Regardless of the care situation, I seek to create a genuine human encounter with the patient even though…	**0.742**				0.491
Regardless of the care situation, I try to encounter each patient as a dignified human being even if…	**0.535**				0.432
In order to ensure good care for my patient…	**0.482**				0.452
2. Moral responsibility					
I bring up for discussion the patient’s right to good care if…			**0.530**		0.585
I participate in care team’s ethical decision-making despite…		**1.008**			0.781
I participate in care team’s ethical decision-making regardless of…		**0.962**			0.813
I bring up my honest opinion concerning even…		**0.362**			0.513
3. Moral integrity					
If someone else* acts professionally dishonestly…			**0.930**		0.692
If someone else* tries to cover up an evident care mistake…			**0.925**		0.722
If someone else* acts unethically…			**0.559**		0.592
I admit my own mistakes…	**0.605**				0.483
I act in accordance with professional ethical principles even if…	**0.535**				0.519
I adhere to professional ethical principles even if…		**0.444**			0.497
I bring up for discussion an ethical problem situation…		**0.413**			0.614
4. Commitment to good care					
If I observe evident shortcomings in someone else’s*…			**0.703**		0.642
If the resources required for ensuring good care are inadequate…			**0.402**		0.473
I do not compromise on my patient’s right to good care even…	**0.563**				0.466
I am even prepared to break prevalent care practices to advocate…				**1.004**	0.752
I bring up for discussion the patient’s right to good care if…				**0.438**	0.469

NMCS: Nurses’ Moral Courage Scale; PCA: principal component analysis.

Someone else*: colleagues, other healthcare professionals, physicians, patient’s next of kin, the patient or the organization where the nurse works.

^a^ Items are listed according to the sub-scales of the NMCS.

**Figure 1.**
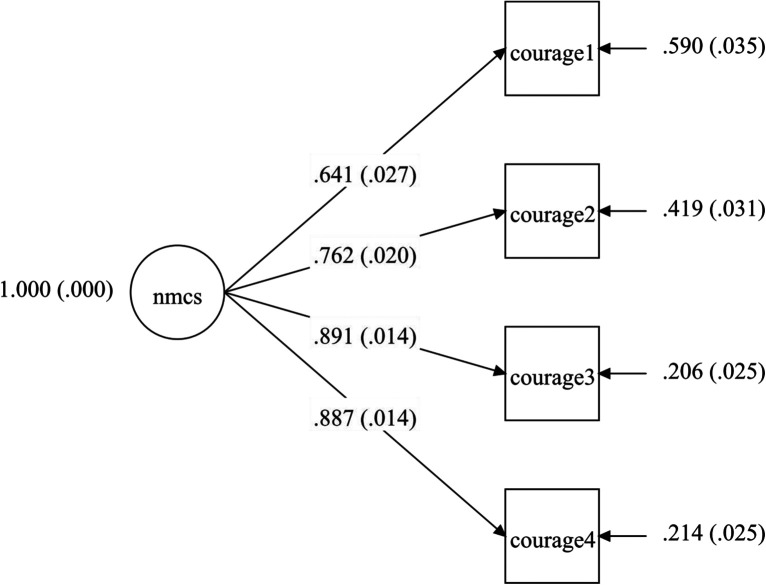
Hypothesized model/Model 1B. NMCS: Nurses’ Moral Courage Scale. Courage 1 = compassion and true presence; Courage 2 = moral responsibility; Courage 3 = moral integrity; Courage 4 = commitment to good care.

The CFA confirmed the construct of the Dutch-language version of the NMCS (n = 559). The construct of the model, which was tested, is described in Figure 1 (hypothesized model/Model 1B). The fit of the model was determined by testing the hypothesized model using structural equation modelling (SEM) (Figure 1, Model 1B) and constructed with maximum likelihood estimations. Several criteria were used to examine the goodness-of-fit of the model, including non-significant chi-square statistics (χ^2^, p value, degree of freedom), CFI, Tucker–Lewis index (TLI), SRMR and RMSEA. The chi-square test is an absolute test of model fit: if the probability value (p value) is below 0.05, the model is rejected. For other indices, the CFI takes into account the model fitting: it may range between 0 and 1, with values near to 1 indicating very good fit.^78^ The CFI value was set in this study at >0.95 or higher. Hu and Bentler (1999)^79^ have recommended a TLI index close to 1.0 and RMSEA values <0.05 as criteria for a fit model. Furthermore, SRMR, which is the most sensitive index to detect mis-specified latent structures or factor/component covariances, was set at 0.08 or lower.^79^ For performing SEM, MPlus version 7.11 was used. All four criteria (CP, CFI, SRMR and RMSEA) confirmed the goodness-of-fit of the model (Table 4).

**Table 4.** Goodness-of-fit indices for the hypothesized model/Model 1B.

Model	χ^2^	p value	df	CFI	TLI	AIC	BIC	SRMR	RMSEA	p ≤ 0.05
1	1.085	0.5813	2	1.000	1.002	3114.50	3166.42	0.004	0.000	0.856

χ^2^, p value: non-significant chi-square statistics; df: degree of freedom; CFI: comparative fit index; TLI: Tucker–Lewis index; AIC: Akaike information criterion; BIC: Bayesian information criterion; SRMR: standardized root mean square residual; RMSEA: root mean square error of approximation.

## Discussion

This methodological study was carried out to validate the Dutch-language 21-item, four-component version of the NMCS. The Dutch-language version of the NMCS proved to be valid and reliable. The scale’s total internal consistency reliability using Cronbach’s alpha coefficient was high (0.91) corresponding with the original Finnish study (0.93). In both studies, the values exceeded the recommended minimum value of 0.7 for a new scale. In the four components, the Cronbach’s alpha values were slightly lower than those in the original Finnish scale, but well acceptable, the differences ranging from 0.01 to 0.05.^14,77^

The forced four-component PCA confirmed the four-component structure of the original scale to be valid also in the Belgian data explaining 58.1% of the variance in the NMCS. CFA based on goodness-of-fit indices provided evidence for construct validity (the hypothesized model fit). In both Belgian and Finnish validation studies, goodness-of fit indices for the hypothesized models/Model 1 and Model 1B fell within acceptable values.^14^ Furthermore, the use of a comparable sample of nurses working in a variety of speciality care settings as in the Finnish study supported the stability of the structure.

However, although the CFA and its sufficiently high Cronbach’s alpha values supported the four-component construct of the NMCS in the Belgian data, attention should be paid to the forced four-component PCA and its item distribution into the components. The PCA showed that nearly two thirds of the items grouped into their components as in the hypothesized model/Model 1B. The remaining eight items seemed to group to a different component than in the hypothesized model/Model 1B. However, the reliance solely on the statistical calculations should be considered with caution, since each item in terms of its theoretical content may not necessarily correspond with the statistically indicated component. Researcher’s theoretical familiarity with the concept and its dimensions is also important in locating items into the components of the hypothesized model. But, in future studies focusing on the NMCS validation, it would be justified also to compute exploratory factor analysis/PCA letting the items group freely to see whether the hypothesized model of the scale needs further development.

Prior to the development of the NMCS,^14^ Dinndorf-Hogenson^70^ had developed the Moral Courage Questionnaire for Nurses (MCQN) to measure nurses’ moral courage focusing strictly on perioperative nurses in response to a specific threat to patient safety,^70,71^ thus not lending the MCQN to be used as a generic measure of nurses’ moral courage. Furthermore, to the present knowledge, the MQCN has not been psychometrically validated. Therefore, its use even as a criterion validity measure in the development of the NMCS was not relevant.^76^

However, referring to various reasons described in the background section of the present study, there was a need to develop more generic instruments which would more extensively cover the concept of moral courage in nursing contexts. As far as we know, the NMCS is the first generic, validated scale to measure nurses’ moral courage and thus, yet in the early state of its development. The theoretical basis of the scale is on a concept analysis related to studies on moral courage in nursing context.^9^ At the time of scale development, research on moral courage in nursing was scarce. Since then, interest in the concept of moral courage has increased as indicated in the growing number of studies and theoretical literature on the subject. Considering the prospects of the development of the NMCS, it would be important also to analyse the recent research and theoretical literature on moral courage and consider whether it could provide new knowledge to better understand the dimensions of this abstract concept in nursing context. This knowledge could be used to further strengthen the conceptual basis of the scale.

Although moral courage is seen as a personal characteristic, courageous action is also dependent on contextual factors, such as the situation itself needing moral courage,^18^ ethical climate of the work environment,^63^ organizational leadership practices,^60^ hierarchal power structures and given support ^6,8,38,80,81^ as well as nursing education and student supervision.^53,82^ These factors should be further studied to understand also their impact on morally courageous behaviour and consider whether inclusion of contextual elements in the scale and formulation of its items should be considered.

However, the close similarity of mean scores between the total scale and the four components in the Belgian and Finnish studies reflects the scale’s suitability to measure nurses’ moral courage in speciality care working environments. It also suggests that the items of the scale seem to apply in a slightly different cultural context in Europe, although further validation studies are needed in varying nursing care environments and care cultures to confirm the scale’s generic applicability to measure nurses’ moral courage.

### Limitations and strengths of the study

There are some limitations to this study. The moderate response rate can be seen as a limitation, but the number of responses allowed the use of statistical methods needed for the scale’s reliability and validity assessments. Based on the statistical power analysis, also the number of participating nurses was adequate, but the study was carried out in only two hospitals. Nevertheless, the data covered a representative sample of nurses working in a large variety of different kinds of medical fields and wards requiring high professional competence in demanding care situations. These care settings also corresponded with the original Finnish data, which could be considered a strength, and consequently confirming the validity of the NMCS to be used with nurses representing tertiary level care but equally well also on less demanding levels of nursing care. Therefore, in translating and validating the NMCS in other countries and care contexts, also the scale’s cultural validation in each country is of paramount importance in relation to the organization of each country’s healthcare system, nursing education and cultural characteristics in general.^83,84^

### Implications and further research

The results of this study and the original Finnish study offer a basis for successive validity verifications in other countries, care cultures and contexts.^85^ Successive validity verification studies might indicate need for further development of the instrument for it to better function in different care cultures and contexts. Further analyses of recent research and theoretical conceptual basis of moral courage in nursing are also relevant. Knowledge gained from further studies of moral courage can be used to identify gaps as well as strengths in nursing students’, nurses’ and nurse leaders’ moral courage and their readiness to act morally courageously. The use of valid and reliable instruments helps in planning educational programmes and interventions for basic and continuing nursing ethics education to advance and strengthen nurses’ ethical competence in demanding situations of moral conflict. Valid and reliable instruments are also needed to produce evidence-based data for harmonization of nursing ethics education.^13,65^ Moral courage is also a crucial element in nurse leadership and management. Measured knowledge of nurse leaders’ moral courage is a good means to assess their moral value base and integrity in leadership and in promoting ethically high-quality care.^64^ Furthermore, along with globalization and human mobility, many health issues have become global. As the largest group of healthcare workers, nurses are in a pivotal role in responding to global healthcare needs. To fulfil this role, nurses need moral courage to provide quality and safe care to their patients and to protect them from even fatal consequences of many health problems. The recent COVID-19 pandemic as a drastic example has shown nurses’ need to overcome fear and to act morally courageously in the face of human vulnerability in relation to their patients, and themselves.^86^ Measurement and generalizable research findings of nurses’ moral courage can contribute to developing means to enhance nurses’ moral courage in responding to global healthcare needs also in the future.

## Conclusion

The Dutch-language, 21-item, four-component version of the NMCS is a reliable and valid instrument to measure nurses’ self-assessed moral courage in speciality care nursing environment in Flanders, Belgium. Further validation studies in other countries and languages as well as including nurses working in various other healthcare environments would provide evidence-based knowledge of nurses’ moral courage. This knowledge could be used in advancing nurses’ ethical competence as autonomous and courageous moral agents in research, practice and leadership and in developing nursing ethics education in the EU and beyond.^27,64^
